# What makes a good quality indicator set? A systematic review of criteria

**DOI:** 10.1093/intqhc/mzab107

**Published:** 2021-07-20

**Authors:** Laura Schang, Iris Blotenberg, Dennis Boywitt

**Affiliations:** Department of Methodology, Federal Institute for Quality Assurance and Transparency in Health Care (IQTIG), Katharina-Heinroth-Ufer 1, Berlin 10787, Germany; Department of Methodology, Federal Institute for Quality Assurance and Transparency in Health Care (IQTIG), Katharina-Heinroth-Ufer 1, Berlin 10787, Germany; Department of Methodology, Federal Institute for Quality Assurance and Transparency in Health Care (IQTIG), Katharina-Heinroth-Ufer 1, Berlin 10787, Germany

**Keywords:** indicator set, criteria, content validity, MeSH: health care quality indicators

## Abstract

**Background:**

While single indicators measure a specific aspect of quality (e.g. timely support during labour), users of these indicators, such as patients, providers and policy-makers, are typically interested in some broader construct (e.g. quality of maternity care) whose measurement requires a set of indicators. However, guidance on desirable properties of indicator sets is lacking.

**Objective:**

Based on the premise that a set of *valid indicators* does not guarantee a *valid set* of indicators, the aim of this review is 2-fold: First, we introduce content validity as a desirable property of indicator sets and review the extent to which studies in the peer-reviewed health care quality literature address this criterion. Second, to obtain a complete inventory of criteria, we examine what additional criteria of quality indicator sets were used so far.

**Methods:**

We searched the databases Web of Science, Medline, Cinahl and PsycInfo from inception to May 2021 and the reference lists of included studies. English- or German-language, peer-reviewed studies concerned with desirable characteristics of quality indicator sets were included. Applying qualitative content analysis, two authors independently coded the articles using a structured coding scheme and discussed conflicting codes until consensus was reached.

**Results:**

Of 366 studies screened, 62 were included in the review. Eighty-five per cent (53/62) of studies addressed at least one of the component criteria of content validity (content coverage, proportional representation and contamination) and 15% (9/62) addressed all component criteria. Studies used various content domains to structure the targeted construct (e.g. quality dimensions, elements of the care pathway and policy priorities), providing a framework to assess content validity. The review revealed four additional substantive criteria for indicator sets: cost of measurement (21% [13/62] of the included studies), prioritization of ‘essential’ indicators (21% [13/62]), avoidance of redundancy (13% [8/62]) and size of the set (15% [9/62]). Additionally, four procedural criteria were identified: stakeholder involvement (69% [43/62]), using a conceptual framework (44% [27/62]), defining the purpose of measurement (26% [16/62]) and transparency of the development process (8% [5/62]).

**Conclusion:**

The concept of content validity and its component criteria help assessing whether conclusions based on a set of indicators are valid conclusions about the targeted construct. To develop a valid indicator set, careful definition of the targeted construct including its (sub-)domains is paramount. Developers of quality indicators should specify the purpose of measurement and consider trade-offs with other criteria for indicator sets whose application may reduce content validity (e.g. costs of measurement) in light thereof.

## Introduction

Health care quality indicators serve to enable their users—such as patients, providers and policy-makers—to make informed decisions based on the quality of care [1–3]. While single indicators measure specific aspects of quality [4], users of these measures are frequently interested in some broader construct. For instance, single indicators may measure the provision of smoking cessation advice or timely support during labour [5]. However, it is the quality of community-based maternity care that would be of interest to patients (e.g. when choosing a provider) or policy-makers (e.g. for accountability purposes) [5, 6]. Since health care quality is multidimensional [7–9] and providers may perform relatively well on some aspects of care, but less so on others [10], multiple indicators are needed to measure constructs such as ‘quality of community-based maternity care’. Conclusions about such constructs thus depend on the properties not only of single indicators but also of the indicator set as a whole [11–14].

So far, however, recommendations for developing quality indicators focus primarily on the criteria for single indicators, such as the validity, reliability and feasibility of an indicator [see e.g. 4, 15–22]. In contrast, guidance on desirable properties of indicator sets is lacking [13, 23].

**Figure 1:**
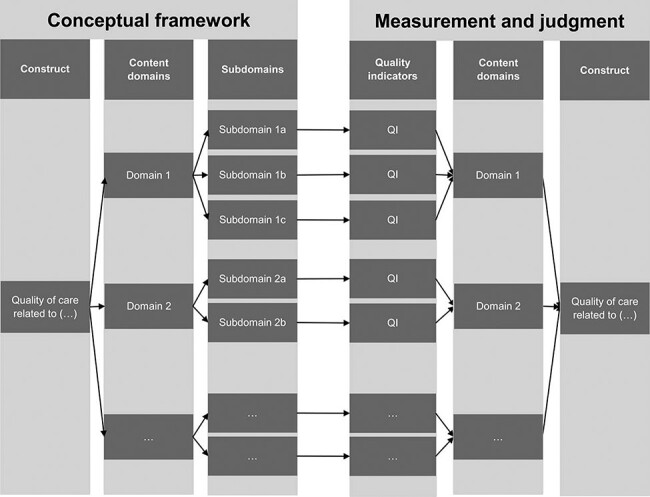
Illustration of content validity using the Brunswik lens model (24–26, own display): The construct of interest (‘what’ to measure) may be quality of care regarding a specific sector, service area or another topic. Content domains and subdomains structure the targeted construct, for instance, in terms of quality dimensions, the care pathway, policy priorities or other domains (see Table 2). The content domains and subdomains thus form the conceptual framework guiding the selection of indicators. A content-valid indicator set covers the relevant content domains and subdomains, assures proportional representation and does not contain irrelevant content (see Table 1). Thus, a content-valid indicator set ensures that conclusions about the targeted construct based on measurement results (see panel on the far right) are valid conclusions about the targeted construct according to the conceptual framework (see panel on the far left; see [28, 30]).

To address this gap, the ‘lens model’ [24–26] provides a helpful starting point: Accordingly, indicators serve as ‘cues’ forming the ‘lens’ through which users of measurement results ‘view’ the targeted construct (see Figure 1). If the ‘cues’ do not represent the construct in a valid fashion, conclusions about the construct may be misguided. Therefore, we propose that content validity constitutes an important property of indicator sets. Generally, assuring content validity of an indicator set means ensuring that the content of the assessment instrument adequately reflects the targeted construct [27–29]. There are three main threats to the content validity of an indicator set: omission of relevant indicators, overrepresentation of indicators for some aspects of care and inclusion of irrelevant indicators. These threats reduce the content validity of the set and, ultimately, limit the quality of conclusions one can draw about the targeted construct based on measurement results [e.g. 28, 30]. As such, content validity provides the theoretical yardstick to confirm—or refute—concerns that existing indicator sets often seem imbalanced [23, 31–33].

Given the current lack of guidance on the criteria for indicator sets [13, 23], the aim of this paper is to take stock of the criteria addressed so far in the peer-reviewed health care quality literature. Since we deem content validity a desirable property of indicator sets, our first research question is: to what extent do studies address the content validity of indicator sets? Second, to obtain a complete inventory of criteria, we ask what additional criteria of indicator sets exist in the health care quality literature. We discuss our results with the aim of providing guidance for those tasked with developing indicator sets.

## Methods

### Search strategy

We systematically searched the databases Web of Science, Medline, Cinahl and PsycInfo on 21 May 2021. To obtain a comprehensive overview of the field, we used the broad search term ‘indicator set’ without any filters or limits. Additionally, we searched the reference lists of included studies.

### Eligibility criteria

#### Inclusion criteria

Studies were eligible for inclusion if they addressed the criteria for indicator sets (defined as desirable properties that can only be assessed at the level of the set [13, 23]), were published in a peer-reviewed journal and focused on health care quality.

#### Exclusion criteria

We excluded studies without full text available and those not written in English or German.

### Study selection

Two authors (L.S. and I.B.) independently screened all titles, abstracts and potentially relevant articles retrieved for full-text review. They resolved any doubts about the eligibility of studies through discussion until consensus was reached.

### Data extraction

Following qualitative content analysis (QCA), we developed a coding scheme with definitions and exemplars for all codes [34, 35], which we used to extract information from each included study. We developed codes in two ways. First, following directed QCA, we used existing theory to develop codes [34, 36]. Since content validity comprises three component criteria—content coverage, proportional representation and contamination [28, 37] (for definitions, see Table 1)—we used these to derive codes deductively.

**Table 1:** Criteria of content validity: definition, exemplar and frequency in included studies

Criteria of content validity	Definition	Exemplar	% of included studies (*N* = 62)
Content coverage	Degree to which the set covers the content domains [30, 37, 53]		71% (44/62)
Breadth	Degree to which the set covers *all relevant content domains*	*‘* *First, potential quality indicators for each dimension of health care to be covered were defined* *.’* *[* *60* *]*	56% (35/62)
Depth	Degree to which the set covers *a specific content domain (and its subdomains)* properly	*‘* *Dimensions or subdimensions that were not properly covered were identified, and literature had to be further reviewed to identify indicators covering properly these areas* *.’ [12]*	15% (9/62)
Not specified	Degree of content coverage, no specification concerning breadth or depth	*‘[…]* *do you think the proposed indicator set presents a complete picture of [e.g., attitudes to aging] in Ireland* *?’* *[* *61* *]*	15% (9/62)
Proportional representation	Number of indicators in each domain matches the importance of the respective domain in the construct [28, 37, 53]	*‘* *We found large differences in the degree to which the dimensions of quality were represented by the identified indicators […] we found safety and effectiveness dominated over other dimensions […] The dimension of patient-centredness, which is acknowledged to be underdeveloped, attracted few indicators. […] [* *32* *]*	31% (19/62)
Contamination	The set does not contain irrelevant indicators [30, 37, 53]	*‘* *Relevance to medication-related quality of care needs for Australian residential aged care (…) Presence of indicators which address one or more of the pre-determined six medication-related attributes is shown* *(…)’ [62]*	50% (31/62)
Σ	Studies addressing at least one of the three criteria		85% (53/62)
Σ	Studies addressing all three criteria of content validity		15% (9/62)

Second, because generally no unified definitions of criteria for indicator sets exist [13, 23], we inductively developed codes in accordance with conventional QCA [34]. Thus, two authors (L.S. and I.B.) read all documents and, in iterative discussions with D.B., determined codes by identifying desirable characteristics of indicator sets from the studies themselves [34, 38, 39]. To achieve this, we examined definitions and procedures adopted by the studies. We did not code mere labels or adjectives whose meaning remained unclear (e.g. ‘comprehensive’, ‘wide scope’). Instead, we coded text segments only if the authors described what they meant or did to assure ‘good’ indicator sets. In addition, we extracted information on the construct targeted by the respective study (e.g. diabetes care) and on the domains (e.g. quality dimensions) selected by the authors to assess content validity.

To ensure a consistent understanding of the codes, two authors (L.S. and I.B.) independently coded and compared the results of an identical sample of articles. Subsequently, both authors repeated this process for all articles using the analysis software MAXQDA. Any conflicts in coding were reconciled through discussion until consensus was reached.

### Data synthesis

To synthesize the data in relation to our research questions, we tabulated the absolute and relative frequencies of the criteria and the domains identified from all included studies.

## Results

**Figure 2:**
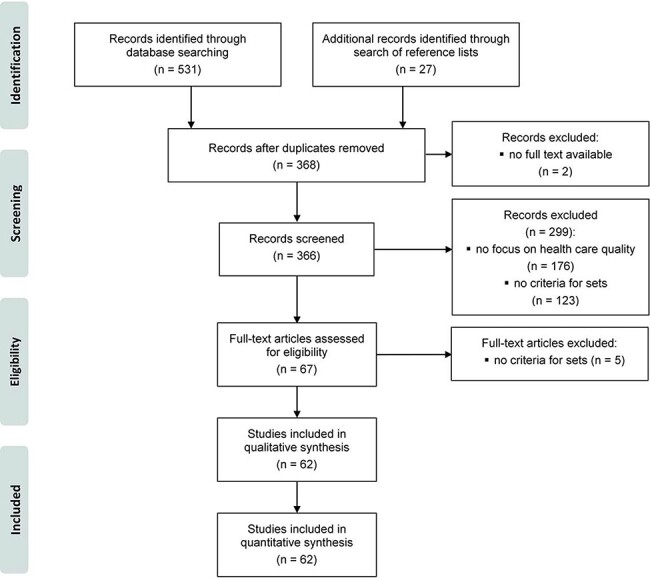
Study selection process.

Of 531 studies identified through database searching and 27 studies identified through the search of reference lists, we ultimately included 62 studies (Figure 2; for details see Supplementary Appendix 1 and Appendix 2). The studies addressed a variety of constructs, including, amongst others, quality of hospital care [12], quality of primary care [40], quality of mental health care [41] or quality of community-based maternity care [5] (for details on all studies, see Supplementary Appendix 2).

In 90% (56/62) of the studies, authors structured the construct they intended to measure in content domains, such as quality dimensions, policy priorities or elements of the care pathway (Table 2). Frequently, studies also referred to the coverage of different measurement domains (Table 2).

**Table 2:** Domains for structuring health care quality constructs

Content domains	Definition	Exemplar	% of included studies (*N* = 62)
Tailored domains	The set addresses tailored domains deemed important according to a specific framework	*‘[…]* *we conceived a conceptual framework […] of high quality palliative care consisting of several domains: 1) physical, 2) psychological, social and existential, 3) information, communication, planning and decision making with patients, 4) with family and 5) with other carers, 6) type of care, 7) coordination and continuity, 8) support of friend or family carers and 9) structure of care* *.’* *[* *45* *]*	47% (29/62)
Quality dimensions	The set addresses generic quality dimensions, e.g. based on [63]	*‘[…]* *ensure that selected indicators addressed all dimensions of quality (safe, effective, patient centred, timely, efficient and equitable* *).’* *[* *64* *]*	37% (23/62)
Care pathway	The set addresses service needs along the care pathway	*‘* *During this study, a set of 52 quality indicators was developed to reflect the entire pathway of colorectal cancer care* *.’* *[* *57* *]*	19% (12/62)
Policy priorities	The set addresses national/regional health policy priorities/goals	*‘* *This paper summarizes the major policy goals (which are the cornerstones of mental health reform) and suggests a series of high-level indicators to assess performance toward achieving these goals* *.’* *[* *41* *]*	16% (10/62)
Sectors	The set addresses different health care sectors (e.g. inpatient, outpatient)	*‘[…]* *it can be expected that in the future a stronger focus will be expected by financers and users to address longer-term and sector-wide performance assessments* *.’ [65]*	15% (9/62)
Service areas	The set addresses different service areas/specialties (e.g. cardiology and gynecology)	*‘* *In Germany, hospital quality indicators focused almost entirely on the safety and medical effectiveness of a few, largely surgical, interventions* *.’* *[* *31* *]*	13% (8/62)
Information needs of stakeholders	The set addresses specific information needs of stakeholders	*‘* *In ECHI, this has been emphasized by the definition of “* *user-windows* *.”* *These are subsets from the overall indicator list, each of which should reflect a specific user’s requirement or interest* *.’* *[* *66* *]*	15% (9/62)
Health care needs over the life cycle	The set addresses health care needs over the life cycle (e.g. stay healthy and get better)	*‘* *A high-quality and safe healthcare system should provide quality care a cross each of the stages at which persons access it: to stay healthy, to get better, to live with illness or disability and to cope with end of life* *.’* *[* *64* *]*	5% (3/62)
Σ	Studies using (any) content domains to structure the construct		90% (56/62)
**Measurement domains**	**Definition**	**Exemplar**	**% of included studies(*N* = 62)**
Structure, process, outcome	The set addresses specific measurement domains according to [67]	*‘* *The ideal balance between structural, process and outcome indicators in quality measurement remains to be elucidated* *.’* *[* *32* *]*	68% (42/62)
Σ	Studies using measurement domains and content domains		58% (36/62)
Σ	Studies using only measurement domains to structure the construct		10% (6/62)

### Research question 1: to what extent do studies address the content validity of indicator sets?

Overall, while only 19% (12/62) of the studies in our review used the term ‘content validity’, 85% (53/62) of the studies addressed at least one of its component criteria. Only nine studies (15%) addressed all three criteria (Table 1).

#### Content coverage

Seventy-one per cent (44/62) of studies referred to the criterion ‘content coverage’ (Table 1). While more than half of all studies (35/62) addressed content coverage in terms of the ‘breadth’ of content domains covered, 15% (9/62) additionally referred to the ‘depth’ of coverage of a specific content domain (with respect to its subdomains).

#### Proportional representation

Proportional representation was addressed by about a third of the studies (19/62); typically, by commenting on unequal numbers of indicators across different quality dimensions (see exemplar in Table 1). Some studies pre-specified a particular number of indicators for each domain in order to ensure proportional representation of all content domains in the indicator set [e.g. 33, 42].

#### Contamination

Half of the studies (31/62) referred to avoiding the contamination of the indicator set by including indicators only if they were relevant for the targeted construct (Table 1).

### Research question 2: what additional criteria of indicator sets exist in the health care quality literature?

#### Additional substantive criteria

We identified four additional substantive criteria of indicator sets from the included studies (Table 3). Studies concerned with ‘costs of measurement’ frequently addressed the burden of data collection imposed on providers (see exemplar, Table 3). While several studies referred to the ‘size’ of the set, this criterion was frequently introduced as a means to an end, e.g. to reduce costs of measurement (by reducing the number of indicators) [e.g. 22, 43], to enhance content coverage (by increasing the number of indicators) [42] or to promote proportional representation (by aiming for a specified number of indicators in each content domain) [33, 44]. With respect to the criterion ‘prioritization’, studies typically used a ranking or rating procedure to identify the ‘most important’ or ‘essential’ indicators. Some studies also mentioned avoiding redundancy as a criterion.

**Table 3:** Additional criteria for indicator sets: definition, exemplar and frequency in included studies

Substantive criteria	Definition	Exemplar	% of included studies (*N* = 62)
Cost of measurement	Costs associated with measuring the set as a whole (related to, e.g. data collection, analysis and reporting)	*‘* *Application of the new indicator set was found to be feasible by participating physicians and hospitals. Median time to document the required information for 1 patient was 5 minutes* *.’* *[* *60* *]*	21% (13/62)
Avoid redundancy	Additional indicators do not duplicate existing indicators	*‘[…]* *if existing projects collect similar indicators with slightly different definitions, which could result in a high burden of data collection and could impact negatively on the motivation* *.’* *[* *65* *]*	13% (8/62)
Size	The set consists of an appropriate/a specified number of indicators	*‘* *The goal was to form a concise measurement set of approximately 10 indicators, although it was recognized that the final number of indicators would be responsive to the concerns of both comprehensiveness and brevity* *.’* *[* *42* *]*	15% (9/62)
Prioritization	The set includes the ‘most important’ or ‘essential’ indicators for the purpose of assessment	*‘* *The purpose of the CUP [Clinical User Panel] process was to select the indicators that were the most clinically important and usable* *.’* *[* *42* *]*	21% (13/62)
**Procedural criteria**	**Definition**	**Exemplar**	**% of included studies (*N* = 62)**
Consider assessment purpose	The set is developed with the assessment purpose in mind	*‘* *Selective contracting requires comparative information, because health insurers want to contract the best and/or the cheapest providers. Pay-for-performance contracts may require information about current performance* *[…]’* *[* *68* *]*	26% (16/62)
Develop/use conceptual framework	The set is developed based on a conceptual framework	*‘[…]* *indicator development should proceed in a systematic fashion, targeting areas where the need is greatest, and have described a framework to assist with this aim* *.’ [32]*	44% (27/62)
Stakeholder involvement	Stakeholder groups are involved in the development process		69% (43/62)
Provider involvement	Provider groups are involved in the development process	*‘* *Selected experts from high-volume DBS [Deep Brain Stimulation] centers across Germany were invited to join the QI Board for the development of evidence-based QIs* *.’ [69]*	48% (30/62)
Patient involvement	Patient groups are involved in the development process	*‘* *However, it has also become clear that particular attention should be given to the participation of patient/consumer organisations* *.’* *[* *68* *]*	39% (24/62)
Other	Other groups (e.g. researchers and purchasers) are involved in the development process	*‘* *Within each working group clinicians, epidemiologists and experts in quality management were represented* *.’* *[* *60* *]*	44% (27/62)
Transparency of development process	Methods and limitations are transparently presented	*‘[…]* *a variety of studies have focused on quality indicators for palliative care, the methods found in the literature by which indicators were developed were not always clearly presented* *[…].’* *[* *45* *]*	8% (5/62)

#### Procedural criteria

Several studies also pointed out the desirable properties of the process of developing indicator sets (Table 3). While the rationale behind these procedural criteria often remained unclear, in several studies, they appeared to serve as a means to assure content validity. Several studies developed a framework that was then used to map indicators and thus assure content coverage [5, 45, 46]. Early involvement of stakeholders, in turn, served to define the construct and identify the relevant content domains by eliciting aspects considered important from the perspectives of patients and providers [e.g. 5, 33]. During the process of indicator selection, stakeholders were frequently involved to ensure content coverage [e.g. 5, 12] and prevent contamination of the set [e.g. 40, 47]. Some studies also emphasized the need to consider the assessment purpose when developing indicator sets and to ensure transparency about methods and limitations (Table 3).

## Discussion

### Statement of principal findings

Regarding our first research question—the extent to which studies in the health care quality literature address content validity as a criterion for indicator sets—three principal findings emerge. First, while 85% (53/62) of the studies addressed at least one of the component criteria of content validity (content coverage, proportional representation, or contamination), suggesting that most studies consider (components of) content validity important, only 15% (9/62) addressed all of its component criteria. Second, our review revealed that several authors distinguished between the ‘breadth’ and ‘depth’ of content coverage. Third, we found that authors used various content and/or measurement domains to structure the targeted construct in order to provide a framework for assessing content validity.

Regarding our second research question, we further identified four substantive criteria and four procedural criteria. Among the former, costs of measurement and prioritization of ‘essential’ indicators were addressed most frequently (each by 21% [13/62] of the included studies). Among the latter, several studies emphasized the importance of defining or using a conceptual framework (44% [27/62]) and stakeholder involvement (69% [43/62]).

### Strengths and limitations

Our review is, to our knowledge, the first review of criteria for indicator sets in the health care quality literature. These criteria are an inventory of what previous studies have considered important properties of indicator sets. As such, the review offers a valuable guide for those tasked with developing indicator sets and for further research on this topic. Second, with our analytic approach, we went beyond the frequently inconsistent terminology in the studies and examined instead what the authors recommended or did to obtain ‘good’ indicator sets. This enabled us to offer a taxonomy of criteria and, based on consistent definitions, to report their frequencies in the studies included.

Our study has limitations. First, while our review was extensive in that it covered four scientific databases using broad search terms, we focussed on the peer-reviewed health care quality literature and did not examine in detail other fields (e.g. sustainability and education). From the non-health studies examined, however, we identified no additional criteria [11, 48, 49]. Second, searches of the grey literature might have yielded additional criteria. However, including searches of grey literature in a systematic review also entails several limitations, such as poor methodological reproducibility, missing citation information and varying indexing and search functionalities of Web-based search engines and repositories [50]. Third, QCA always involves some subjectivity in coding [34]. However, we took several steps to enhance the trustworthiness of the results, including the use of a coding scheme, coder training to ensure consistent implementation of the scheme, independent coding by two reviewers and comparison of all conflicts until consensus was reached [35, 39]. We are therefore convinced that our results provide a credible account of the reviewed studies.

### Interpretation within the context of the wider literature

Typically, users of measurement results want to draw valid conclusions about some broader construct (such as a provider’s quality of primary care [40] or quality of mental health care [41], as in some of the studies in our review). In these cases, an exclusive emphasis on the methodological quality of single indicators is insufficient: it might result in incomplete coverage, overrepresentation of indicators for some aspects of care and/or superfluous indicators [11]. Because each component criterion of content validity helps to remedy one of these threats [e.g. 28], an indicator set becomes more valid when all three component criteria are assured [e.g. 28, 30]. Thus, our finding that only 15% (9/62) of the included studies sought to assure all three component criteria suggests the need for a stronger emphasis on content validity for developers of indicator sets.

Health care quality constructs are frequently conceptualized in terms of multiple levels, with several domains and subdomains (12, 13, 45; see also Figure 1). Thus, the distinction between the ‘breadth’ and ‘depth’ of content coverage we found in several studies seems important for quality indicator sets. While an indicator set may address all relevant content domains (thus achieving high ‘breadth’), the ‘depth’ to which each of these domains is covered also influences the degree to which an indicator set measures what it purports to measure [13]. Therefore, it seems important to assess both the ‘breadth’ and ‘depth’ of content coverage of quality indicator sets.

Content validity is assessed with reference to content domains [28, 30]. Therefore, careful development of the (sub-)domains of the targeted construct represents the crucial first step to obtain a valid indicator set [28, 29]. Our finding that more than two thirds (42/62) of the reviewed studies employed Donabedian’s generic measurement domains to assess indicator sets may reflect the enduring debate in the literature about the merits and demerits of structure, process and outcome indicators [51, 52]. These measurement domains, however, are not helpful for structuring the construct. For instance, patient safety of primary care can be measured with structure, process and outcome indicators, but this would not ensure the coverage of other quality dimensions of the construct ‘quality of primary care’ such as effectiveness and responsiveness [13]. Therefore, we caution against using measurement domains as a substitute for actual content domains. Instead, we suggest, the development of the content domains should be driven by the quality objectives regarding the targeted construct [53, 54].

Our findings also reflect long-standing tensions between maximising insights gained from measurement and minimising costs to obtain these insights [11, 55]. While ‘comprehensive’ measurement of all aspects of health care quality has been deemed an unrealistic ambition [13, 56], it is important to emphasize that assuring content validity does not entail measuring ‘everything’. Rather, it involves making explicit the content domains that are relevant for the targeted construct and the degree to which an indicator set represents these domains [27, 28]. The criterion ‘prioritization’ identified in the literature seems premised on the notion that some indicators are more important to the targeted construct than others. The consequent exclusion of (relevant) indicators reduces, however, content validity and limits the ability to draw conclusions about the targeted construct [27, 28]. Similar trade-offs arise with the criterion ‘size’: Unless a relatively narrow construct such as preoperative management in colorectal cancer care [57] is targeted, it is difficult to achieve a highly content-valid indicator set with very few indicators [11, 48]. Yet, a large number of indicators does not guarantee high content validity [11], for instance, when not all relevant content domains are covered.

### Implications for policy, practice and research

The component criteria of content validity help with assessing whether conclusions based on a set of indicators are valid conclusions about the targeted construct. Those tasked with developing quality indicators should therefore assure the validity of not only single indicators but also of the indicator set as a whole. Developers of quality indicators should specify the purpose of measurement and consider trade-offs with other potential criteria for indicator sets whose application may reduce content validity (e.g. costs of measurement and prioritization) in light thereof.

To develop a valid indicator set, careful definition of the targeted construct, including its (sub-)domains, is paramount: Since content validity can only be assessed in relation to a conceptual framework [27, 28], the indicator set can only be as good as the chosen framework. The conceptual framework should serve as a mapping tool to select indicators and to signal gaps in content coverage [11, 21, 58, 59]. Building on the finding that the indicator set can only be as good as the content domains specified, future research should examine how different purposes of quality measurement, such as accountability and improvement [3], influence how the targeted construct should be conceptualized.

## Conclusions

Based on the premise that a set of ‘valid indicators’ does not guarantee a ‘valid set’ of indicators, this review takes stock of existing criteria for indicator sets in the health care quality literature with a focus on content validity. These criteria can guide the process of developing indicator sets and, by complementing the assessment of single indicators, support patients, providers and policy-makers in making informed decisions based on the results of quality measurement.

## Supplementary Material

mzab107_Supp

## Data Availability

Data on all reviewed studies are incorporated in Supplementary Appendix 2.
