# Supplementary material for: What makes a good quality indicator set? A systematic review of criteria
Source: Int J Qual Health Care. 2021 Jul 20;33(3):mzab107. doi: 10.1093/intqhc/mzab107 (PMC8325455; doi:10.1093/intqhc/mzab107)
Supplement: mzab107_Supp [file mzab107_supp.zip › Supplementary.docx]

## Supplementary material

Appendix 1. Documentation of search process

1. **Databases and search strategies**

1. Medline

| Interface: Ovid  Date of Search: May 21, 2021  Number of hits: 168 |
| --- |
| Search term: “indicator set”  No filters and limits were used. |

2. Cinahl

| Interface: Ebsco  Date of Search: May 21, 2021  Number of hits: 108 |
| --- |
| Search term: “indicator set”  No filters and limits were used. |

3. Web of Science

| Interface: webofknowledge.com  Date of Search: May 21, 2021  Number of hits: 229 |
| --- |
| Search term: “indicator set”  No filters and limits were used. |

4. PsycInfo

| Interface: Ovid  Date of Search: May 21, 2021  Number of hits: 26 |
| --- |
| Search term: “indicator set”  No filters and limits were used. |

1. **Full-text articles excluded**

Five articles were excluded after full-text screening, because they did not address criteria for indicator sets:

1. Liu H, Kenji I. Conceptual framework for holistic dialysis management based on key performance indicators. Therapeutic Apheresis and Dialysis. 2013;17(5):532-550.
2. Mourad SM, Nelen WLDM, Hermens RPMG, Bancsi LF, Braat DDM, Zielhuis GA, et al. Variation in subfertility care measured by guideline-based performance indicators. Hum Reprod. 2008;23(11):2493-500.
3. Paquette-Warren J, Tyler M, Fournie M, Harris SB. The Diabetes Evaluation Framework for Innovative National Evaluations (DEFINE): Construct and Content Validation Using a Modified Delphi Method. Canadian journal of diabetes. 2017;41(3):281-96.
4. van der Willik JH, Reijneveld SA, Michaud PA, Kocken P, Jansen D. The Primary Care Indicator Set for Adolescents: the EU MOCHA project: Danielle Jansen. European journal of public health. 2016;26(Suppl. 1):23.
5. van Lieshout J, Nouwens E, Bouma M, Spreeuwenberg C, Wensing M. Consistency of performance indicators for cardiovascular risk management across procedures and panels. Quality & Safety in Health Care. 2010;19(5):e31.

Appendix 2. Study-specific information and results for criteria for quality indicator sets

**Table A.** Information about included studies on quality criteria for indicator sets

| **Reference** | **Country (authors)** | **Aim / targeted construct** | **Domains for structuring the construct** | **Substantive criteria for indicator sets** | **Procedural criteria for indicator sets** |
| --- | --- | --- | --- | --- | --- |
| Ahmed et al., 2020 (1) | Germany, Pakistan | Development of a list of adolescent sexual and reproductive health key indicators for South Asia | - Tailored domains | - Content coverage (breadth) - Contamination | - Stakeholder involvement  (other stakeholders) |
| Alguren et al., 2021 (2) | Sweden, Switzerland, Germany | Analysis and comparison of the content of five registries and two standard sets related to cardiovascular diseases | - Information needs of stakeholders - Quality dimensions - Tailored domains - Measurement domains | - Content coverage (breadth) - Proportional representation | - Develop / use conceptual framework |
| Baars et al., 2010 (3) | The Netherlands | Review on performance indicators in mental health care | - Policy priorities - Quality dimensions - Care pathway - Tailored domains - Measurement domains |  | - Consider assessment purpose - Develop / use conceptual framework |
| Badawi et al., 2015 (4) | Kuwait, Lebanon, USA | Pilot for measuring Type II diabetes care in primary health care centers in Kuwait | - Tailored domains - Measurement domains |  | - Stakeholder involvement (other stakeholders) |
| Beaussier et al., 2020 (5) | France, UK | Comparison of countries’ official indicator sets (England, Germany, France, The Netherlands) used by statutory hospital regulators | - Policy priorities - Service areas - Quality dimensions - Measurement domains | - Content coverage (breadth) - Proportional representation |  |
| Berg et al., 2005 (6) | The Netherlands | Development and implementation of first national, public, obligatory set of hospital performance indicators | - Quality dimensions - Measurement domains | - Cost of measurement | - Consider assessment purpose - Stakeholder involvement (provider, other) |
| Borzecki and Rosen, 2020 (7) | USA | Discussion of strengths and weaknesses of patient safety measurement systems | - Sectors (inpatient and outpatient) - Tailored domains | - Content coverage (breadth) | - Consider assessment purpose |
| Brand et al., 2011 (8) | Australia, USA | Development of aged care quality indicators for acute care hospitals | - Measurement domains | - Proportional representation - Cost of measurement |  |
| Broaddus-Shea et al., 2019 (9) | Switzerland (WHO) | Comprehensive mapping of published indicators for measuring sexual and reproductive health in humanitarian settings | - Service areas - Measurement domains | - Content coverage (breadth + depth) - Proportional representation - Contamination | - Develop / use conceptual framework - Stakeholder involvement (other) |
| Burke and Werner, 2019 (10) | USA | Discussion of future directions for nursing home measures | - Measurement domains | - Proportional representation |  |
| Carinci et al., 2015 (11) | Multi-country | Review and update of the OECD Health Care Quality Indicators (HCQI) project | - Service areas - Health care needs over the life cycle - Sectors (inpatient and outpatient) - Quality dimensions | - Content coverage (breadth + depth) - Contamination - Avoid redundancy | - Consider assessment purpose - Develop / use conceptual framework - Stakeholder involvement (other stakeholders) - Transparency of development process |
| Copnell et al., 2009 (12) | Australia | Identification and classification of indicators measuring quality of hospital care | - Policy priorities - Service areas - Sectors - Quality dimensions - Measurement domains | - Content coverage  (not specified) - Proportional representation | - Develop / use conceptual framework |
| Cramer-van der Welle et al., 2021 (13) | The Netherlands | Development of a relevant set of outcome indicators for lung cancer | - Information needs of stakeholders - Tailored domains | - Content coverage (not specified) - Contamination - Prioritization - Size - Cost of measurement | - Develop / use conceptual framework - Stakeholder involvement (patient, provider, other stakeholders) |
| Dancet et al., 2013 (14) | Belgium, The Netherlands | Development of a quality  indicator set for infertility care | - Quality dimensions - Measurement domains | - Content coverage (breadth) - Proportional representation - Size | - Stakeholder involvement (patient, provider) |
| Delnoij et al., 2010 (15) | The Netherlands | Description of stakeholder involvement in the development of health care quality indicator sets | - Information needs of stakeholders - Quality dimensions |  | - Consider assessment purpose - Stakeholder involvement (patient) |
| De Schreye et al., 2017 (16) | Belgium | Development of an indicator set measuring end-of-life care for people with cancer, chronic obstructive pulmonary  disease or Alzheimer's disease | - Service areas - Tailored domains | - Content coverage (breadth) | - Stakeholder involvement (patient, provider) |
| Döbler & Geraedts, 2018 (17) | Germany | Assessment of the balance of quality indicator sets of external quality assurance | - Quality dimensions | - Content coverage (breadth + depth) - Proportional representation - Contamination | - Consider assessment purpose - Develop / use conceptual framework - Transparency of development process |
| Döbler et al., 2019 (18) | Germany | Call for further research concerning indicator sets and their appropriateness for different assessment purposes | - Policy priorities - Service areas - Sectors - Quality dimensions - Tailored domains - Measurement domains | - Content coverage (breadth + depth) | - Consider assessment purpose |
| Dy et al., 2015 (19) | USA | Development of an indicator set for internal measurement of hospice and palliative care | - Tailored domains - Measurement domains | - Content coverage (breadth) - Contamination - Prioritization - Size | - Consider assessment purpose - Stakeholder involvement (provider, patient, other) |
| Evans et al., 2009 (20) | Australia | Proposition of  a clearly structured process for selecting health care quality indicators at a national and local level | - Health care needs over the life cycle - Sectors - Quality dimensions - Measurement domains | - Content coverage (breadth) - Avoid redundancy | - Develop / use conceptual framework - Consider assessment purpose - Stakeholder involvement (provider) |
| Ewald et al., 2018 (21) | Germany, Spain, Cyprus | Development of a set of Quality Indicators (QIs) to assess paediatric primary care in Europe | - Quality dimensions - Tailored domains - Measurement domains | - Content coverage (breadth) - Contamination - Avoid redundancy | - Stakeholder involvement (provider) |
| Fischer et al., 2016 (22) | The Netherlands | Study the construct validity of national hospital quality  indicator set for hip replacements | - Measurement domains | - Content coverage (breadth) |  |
| Geraedts et al., 2017 (23) | Germany | Discussion of principal quality of care and patient safety research questions and methods | - Quality dimensions - Measurement domains | - Content coverage (breadth) |  |
| Geraedts et al., 2020 (24) | Germany | Development of an indicator set for the evaluation of a regional integrated care programme (Kinzigtal, Germany) | - Health care needs over the life cycle - Sectors - Service areas - Quality dimensions - Measurement domains | - Content coverage (breadth + depth) - Contamination - Avoid redundancy | - Develop / use conceptual framework - Stakeholder involvement (patients, provider, other) |
| Gibney et al., 2019 (25) | Ireland | Measure consensus on a set of positive aging outcome indicators in Ireland | - Policy priorities - Information needs of stakeholders - Tailored domains | - Content coverage (not specified) - Contamination - Prioritization | - Stakeholder involvement (other) - Transparency of development process |
| Groene et al., 2008 (26) | Spain, Denmark | Review of current indicator projects for hospital performance assessment | - Quality dimensions - Sectors | - Cost of measurement - Avoid redundancy | - Stakeholder involvement (patient, provider, other) - Develop / use conceptual framework |
| Grooten et al., 2018 (27) | Belgium, The Netherlands | Test of the content validity of the B3-Maturity Model (addressing integrated care in Europe) | - Tailored domains | - Content coverage (not specified) - Proportional representation - Contamination | - Develop / use conceptual framework - Stakeholder involvement (patient) |
| Haas et al., 2019 (28) | Germany | Presentation of  performance measures of Deep Brain Stimulation  therapy in Parkinson’s disease | - Care pathway - Measurement domains | - Content coverage (breadth) - Proportional representation - Contamination - Cost of measurement | - Develop / use conceptual framework - Stakeholder involvement (provider, other) |
| Hancock et al., 1999 (29) | Canada | Presentation of an initial set of core indicators measuring population health at the community level | - Tailored domains - Measurement domains | - Content coverage ( breadth) | - Consider assessment purpose - Develop conceptual framework - Stakeholder involvement (other) |
| Heuschmann et al., 2006 (30) | Germany | Presentation of performance measures for acute stroke care | - Care pathway - Measurement domains | - Content coverage (breadth) - Proportional representation - Cost of measurement | - Consider assessment purpose - Stakeholder involvement (provider, other) |
| Hillen et al., 2015 (31) | Australia | Review on medication-related quality of care indicators with  respect to application to residential aged care | - Service areas - Tailored domains - Measurement domains | - Content coverage (breadth) - Contamination - Prioritization | - Stakeholder involvement (patient) |
| Hommel et al., 2016 (32) | The Netherlands | Development of quality indicators for optimal perioperative diabetes care | - Care pathway - Measurement domains | - Content coverage (breadth) - Proportional representation - Contamination - Size - Prioritization | - Stakeholder involvement (provider, patient) |
| Iliffe et al., 2016 (33) | UK | Evaluation of the potential of QIs as tools to improve palliative care for people with cancer or dementia in England | - Information needs of stakeholders - Tailored domains |  | - Consider assessment purpose - Stakeholder involvement (provider, patient, other) |
| Johansen et al., 2019 (34) | Norway | Development of an indicator set for rehabilitation of rheumatic and musculoskeletal diseases | - Tailored domains - Measurement domains | - Content coverage (breadth) - Contamination - Cost of measurement | - Stakeholder involvement (provider, patient) |
| Kramers, 2003 (35) | The Netherlands | Proposition of a comprehensive  list of ‘European Community Health Indicators’ | - Policy priorities - Information needs of stakeholders - Tailored domains | - Content coverage (breadth) - Prioritization |  |
| Kringos et al., 2010 (36) | The Netherlands | Development of a Primary Care Monitoring System for application in 31 European countries | - Quality dimensions - Measurement domains | - Content coverage (breadth + depth) - Contamination - Prioritization | - Develop / use conceptual framework - Stakeholder involvement (other) |
| Kuske et al., 2013 (37) | Germany | Review of patient safety indicators | - Sectors (outpatient + inpatient) - Tailored domains - Measurement domains | - Contamination - Prioritization | - Develop / use conceptual framework |
| Leemans et al., 2013 (38) | Belgium | Development of a quality indicator set for palliative care for adults in Flanders | - Tailored domains - Measurement domains | - Content coverage (breadth, depth) - Proportional representation - Contamination - Prioritization | - Develop / use conceptual framework - Stakeholder involvement (provider, patient, other) - Transparency of development process |
| Liu and Itoh, 2013 (39) | Japan | Development of a conceptual framework for hospital performance measurement in Japan | - Quality dimensions - Measurement domains | - Content coverage (not specified) - Contamination - Cost of measurement | - Develop / use conceptual framework - Stakeholder involvement (provider, patient, other) |
| Ludt et al., 2013 (40) | Germany | Development of a set of quality indicators for colorectal cancer care in Germany | - Sectors - Quality dimensions - Tailored domains - Care pathway - Measurement domains | - Content coverage (breadth) - Contamination | - Stakeholder involvement (provider, patient) |
| Mason et al., 2017 (41) | Germany, UK, Switzerland | Development of indicators for community-based rehabilitation | - Tailored domains - Measurement domains | - Prioritization - Cost of measurement | - Develop / use conceptual framework - Stakeholder involvement (other) |
| McCarthy et al., 2008 (42) | UK | Report on properties of data sets relating to breast, colorectal, lung and prostate cancer (England) | - Measurement domains | - Content coverage (breadth) |  |
| McEwan and Goldner, 2002 (43) | Canada | Presentation of indicators to assess performance toward achieving policy goals of mental health reform | - Policy priorities | - Content coverage (breadth) - Contamination - Prioritization - Size | - Develop / use conceptual framework |
| Meyer et al., 2012 (44) | USA | Call for balance and parsimony in quality measurement | - Information needs of stakeholders - Measurement domains | - Proportional representation - Prioritization - Cost of measurement | - Stakeholder involvement (unspecified) |
| Murray et al., 2010 (45) | UK | Development of metrics for monitoring local inequalities in access to maternity care | - Tailored domains - Care pathway | - Content coverage (breadth) | - Stakeholder involvement (provider, patient, other) |
| NHS, 2016 (46) | UK | Presentation of key principles behind developing,  understanding and using indicators | - Policy priorities | - Content coverage (not specified) - Proportional representation - Contamination - Cost of measurement - Prioritization - Size |  |
| Parkinson, 2006 (47) | UK | Identification of a set of national mental health and well-being indicators for Scotland | - Policy priorities - Tailored domains | - Content coverage (not specified) | - Develop / use conceptual framework |
| Riain et al., 2014 (48) | Ireland, UK | Development of a national quality indicator set for Irish general practice | - Tailored domains - Measurement domains | - Contamination - Avoid redundancy | - Stakeholder involvement (provider, patient, other) - Develop / use conceptual framework |
| Schmitt et al., 2013 (49) | Germany | Proposition for the advancement of quality assessment in Germany based on methods of outcomes research | - Measurement domains | - Content coverage (breadth + depth) | - Stakeholder involvement (unspecified) |
| Smith et al., 2007 (50) | USA | Identification of process quality indicators essential  to high-quality, home-based primary care | - Tailored domains - Measurement domains | - Proportional representation - Contamination | - Stakeholder involvement (provider, other) |
| Spaeth-Rublee et al., 2010 (51) | Multi-country | Review of grey literature describing current initiatives that assess the quality of mental health care in 12 countries (Australia, Canada, England, Germany, Ireland, Japan, The Netherlands, New Zealand, Norway, Scotland, Taiwan, US) | - Policy priorities - Quality dimensions - Measurement domains |  | - Consider assessment purpose - Develop / use conceptual framework |
| Stelfox and Straus, 2013 (52) | Canada | Description of an approach for evaluating the value of developing quality indicators | - Information needs of stakeholders - Quality dimensions - Measurement domains | - Content coverage (breadth) | - Develop / use conceptual framework - Stakeholder involvement (patients, provider, other) |
| Strömbeck et al., 2013 (53) | Sweden, The Netherlands | Review of health care quality indicators for rheumatoid arthritis and osteoarthritis | - Information needs of stakeholders - Measurement domains | - Content coverage (breadth) | - Stakeholder involvement (patients, provider, other) |
| Van Damme et al., 2016 (54) | Belgium | Development of a quality of meals and meal service set of indicators for residential facilities for elderly | - Tailored domains - Measurement domains | - Contamination - Avoid redundancy | - Consider assessment purpose - Stakeholder involvement (patient, provider, other) |
| van den Driessen Mareeuw et al., 2017 (55) | The Netherlands | Review of indicators for medical Down syndrome care | - Quality dimensions - Care pathway - Measurement domains | - Proportional representation - Contamination | - Develop / use conceptual framework - Stakeholder involvement (patient, provider, other) |
| van Hulst et al., 2009 (56) | The Netherlands | Development of quality indicators for disease course monitoring in rheumatoid arthritis | - Care pathway - Measurement domains | - Content coverage (breadth) - Contamination | Stakeholder involvement (provider) |
| van Lent et al., 2010 (57) | The Netherlands | Examination of the benchmarking process and the success factors in international specialized cancer centres | - Care pathway | - Contamination | - Develop / use conceptual framework - Stakeholder involvement (provider) |
| Veillard et al., 2005 (58) | Spain, Canada, The Netherlands, USA | Development of a flexible and comprehensive tool for the assessment of hospital performance in Europe | - Quality dimensions - Tailored domains | - Content coverage (breadth + depth) - Contamination - Size | - Consider assessment purpose - Develop / use conceptual framework |
| Voerman et al., 2013 (59) | The Netherlands | Development of a set of quality indicators for public reporting in community-based maternity care | - Care pathway - Tailored domains - Measurement domains | - Content coverage (breadth) - Proportional representation - Contamination | - Consider assessment purpose - Develop / use conceptual framework - Stakeholder involvement (patient, provider, other) |
| Wierenga et al., 2011 (60) | The Netherlands | Development of a set of indicators to measure the quality of in-hospital pharmaceutical care of Dutch elderly patients | - Care pathway - Tailored domains | - Content coverage (not specified) - Contamination - Cost of measurement - Avoid redundancy | - Stakeholder involvement (patient, provider) |
| Wiles et al., 2019 (61) | Australia | Development of a set  of clinical indicators for 21 common paediatric medical conditions | - Quality dimensions - Care pathway |  | - Stakeholder involvement (patient, provider, other) |
| Wollersheim et al., 2007 (62) | The Netherlands | Description of a systematic procedure to achieve qualitatively good clinical indicators and their usage to support improvement | - Measurement domains | - Content coverage (not specified) - Proportional representation - Contamination - Cost of measurement - Size | - Stakeholder involvement (provider) - Transparency of development process |

**References**

1. Ahmed F, Ahmad G, Brand T, Zeeb H. Key indicators for appraising adolescent sexual and reproductive health in South Asia: international expert consensus exercise using the Delphi technique. Global health action. 2020;13:1830555.

2. Algurén B, Jernberg T, Vasko P, Selb M, Coenen M. Content comparison and person-centeredness of standards for quality improvement in cardiovascular health care. PLoS One. 2021;16(1):e0244874.

3. Baars IJ, Evers SMAA, Arntz A, van Merode GG. Performance measurement in mental health care: present situation and future possibilities. The International journal of health planning and management. 2010;25(3):198-214.

4. Badawi D, Saleh S, Natafgi N, Mourad Y, Behbehani K. Quality of Type II Diabetes Care in Primary Health Care Centers in Kuwait: Employment of a Diabetes Quality Indicator Set (DQIS). PLoS One. 2015;10(7):e0132883.

5. Beaussier A-L, Demeritt D, Griffiths A, Rothstein H. Steering by their own lights: Why regulators across Europe use different indicators to measure healthcare quality. Health Policy. 2020;124(5):501-10.

6. Berg M, Meijerink Y, Gras M, Goossensen A, Schellekens W, Haeck J, et al. Feasibility first: Developing public performance indicators on patient safety and clinical effectiveness for Dutch hospitals. Health Policy. 2005;75:59-73.

7. Borzecki AM, Rosen AK. Is there a 'best measure' of patient safety? BMJ: Quality & Safety. 2020;29(3):185-8.

8. Brand CA, Martin-Khan M, Wright O, Jones RN, Morris JN, Travers CM, et al. Development of quality indicators for monitoring outcomes of frail elderly hospitalised in acute care health settings: Study Protocol. BMC: Health Services Research. 2011;11(1):281.

9. Broaddus-Shea ET, Kobeissi L, Ummer O, Say L. A systematic review of monitoring and evaluation indicators for sexual and reproductive health in humanitarian settings. Conflict and health. 2019;13:43.

10. Burke RE, Werner RM. Quality measurement and nursing homes: measuring what matters. BMJ: Quality & Safety. 2019;28(7):520-3.

11. Carinci F, Van Gool K, Mainz J, Veillard J, Pichora EC, Januel JM, et al. Towards actionable international comparisons of health system performance: expert revision of the OECD framework and quality indicators. International Journal for Quality in Health Care. 2015;27(2):137-46.

12. Copnell B, Hagger V, Wilson SG, Evans SM, Sprivulis PC, Cameron PA. Measuring the quality of hospital care: an inventory of indicators. Intern Med J. 2009;39(6):352-60.

13. Cramer-van der Welle CM, van Loenhout L, van den Borne BE, Schramel FM, Dijksman LM. 'Care for Outcomes': systematic development of a set of outcome indicators to improve patient-relevant outcomes for patients with lung cancer. BMJ Open. 2021;11:e043229.

14. Dancet EAF, D'Hooghe TM, Spiessens C, Sermeus W, De Neubourg D, Karel N, et al. Quality indicators for all dimensions of infertility care quality: consensus between professionals and patients. Hum Reprod. 2013;28(6):1584-97.

15. Delnoij DMJ, Rademakers JJ, Groenewegen PP. The Dutch Consumer Quality Index: an example of stakeholder involvement in indicator development. BMC: Health Services Research. 2010;10(1):88.

16. De Schreye R, Houttekier D, Deliens L, Cohen J. Developing indicators of appropriate and inappropriate end-of-life care in people with Alzheimer's disease, cancer or chronic obstructive pulmonary disease for population-level administrative databases: A RAND/UCLA appropriateness study. Palliative medicine. 2017;31(10):932-45.

17. Döbler K, Geraedts M. Ausgewogenheit der Qualitätsindikatorensets der externen Qualitätssicherung nach §136 SGB V. Z Evid Fortbild Qual Gesundhwes. 2018;134:9-17.

18. Döbler K, Schrappe M, Suske S, Schmitt J, Sens B, Boywitt D, et al. Eignung von Qualitätsindikatorensets in der Gesundheitsversorgung für verschiedene Einsatzgebiete - Forschungs- und Handlungsbedarf. Gesundheitswesen. 2019;81(10):781-7.

19. Dy SM, Kiley KB, Ast K, Lupu D, Norton SA, McMillan SC, et al. Measuring What Matters: Top-Ranked Quality Indicators for Hospice and Palliative Care From the American Academy of Hospice and Palliative Medicine and Hospice and Palliative Nurses Association. Journal of pain and symptom management. 2015;49(4):773-81.

20. Evans SM, Lowinger JS, Sprivulis PC, Copnell B, Cameron PA. Prioritizing quality indicator development across the healthcare system: identifying what to measure. Intern Med J. 2009;39(10):648-54.

21. Ewald DA, Huss G, Auras S, Caceres JR, Hadjipanayis A, Geraedts M. Development of a core set of quality indicators for paediatric primary care practices in Europe, COSI-PPC-EU. European Journal of Pediatrics. 2018;177(6):921-33.

22. Fischer C, Lingsma HF, Anema HA, Kievit J, Steyerberg EW, Klazinga N. Testing the construct validity of hospital care quality indicators: a case study on hip replacement. BMC Health Services Research. 2016;16:551.

23. Geraedts M, Drösler SE, Döbler K, Eberlein-Gonska M, Heller G, Kuske S, et al. DNVF-Memorandum III „Methoden für die Versorgungsforschung“, Teil 3: Methoden der Qualitäts- und Patientensicherheitsforschung. Gesundheitswesen. 2017;79(10):e95-e124.

24. Geraedts M, Mehl C, Schmitz J, Siegel A, Graf E, Stelzer D, et al. Entwicklung eines Indikatorensets zur Evaluation der Integrierten Versorgung Gesundes Kinzigtal. Z Evid Fortbild Qual Gesundhwes. 2020;150-152:54-64.

25. Gibney S, Sexton E, Shannon S. Measuring What Matters: Achieving Consensus on a Positive Aging Indicator Set For Ireland. Journal of Aging & Social Policy. 2019;31(3):234-49.

26. Groene O, Skau JKH, Frølich A. An international review of projects on hospital performance assessment. International Journal for Quality in Health Care. 2008;20(3):162-71.

27. Grooten L, Borgermans L, Vrijhoef HJM. An Instrument to Measure Maturity of Integrated Care: A First Validation Study. International Journal of Integrated Care. 2018;18(1):1-20.

28. Haas K, Stangl S, Steigerwald F, Matthies C, Gruber D, Kühn AA, et al. Development of evidence-based quality indicators for deep brain stimulation in patients with Parkinson's disease and first year experience of implementation of a nation-wide registry. Parkinsonism and Related Disorders. 2019;60:3-9.

29. Hancock T, Labonte R, Edwards R. Indicators that Count! Measuring Population Health at the Community Level. Canadian Journal of Public Health. 1999;90(Suppl. 1):S22-6.

30. Heuschmann PU, Biegler MK, Busse O, Elsner S, Grau A, Hasenbein U, et al. Development and Implementation of Evidence-Based Indicators for Measuring Quality of Acute Stroke Care. The Quality Indicator Board of the German Stroke Registers Study Group (ADSR). Stroke. 2006;37(10):2573-8.

31. Hillen JB, Vitry A, Caughey GE. Evaluating medication-related quality of care in residential aged care: a systematic review. SpringerPlus. 2015;4(1):220.

32. Hommel I, van Gurp PJ, Tack CJ, Wollersheim H, Hulscher MEJL. Perioperative diabetes care: development and validation of quality indicators throughout the entire hospital care pathway. BMJ: Quality & Safety. 2016;25(7):525.

33. Iliffe S, Davies N, Manthorpe J, Crome P, Ahmedzai SH, Vernooij-Dassen M, et al. Improving palliative care in selected settings in England using quality indicators: a realist evaluation. BMC palliative care. 2016;15:69.

34. Johansen I, Klokkerud M, Anke A, Børke JB, Glott T, Hauglie U, et al. A quality indicator set for use in rehabilitation team care of people with rheumatic and musculoskeletal diseases; development and pilot testing. BMC Health Services Research. 2019;19:265.

35. Kramers PG. The ECHI project. Health indicators for the European Community. European journal of public health. 2003;13(3 Suppl.):101-6.

36. Kringos DS, Boerma WG, Bourgueil Y, Cartier T, Hasvold T, Hutchinson A, et al. The european primary care monitor: structure, process and outcome indicators. BMC family practice. 2010;11:81.

37. Kuske S, Maass C, Weingärter V, Pöhlmann S, Schrappe M. Patient-safety indicators: a systematic review, criteria-based characterization and prioritization. Journal of Public Health. 2013;21(2):201-14.

38. Leemans K, Cohen J, Francke AL, Stichele RV, Claessen SJJ, Block LVd, et al. Towards a standardized method of developing quality indicators for palliative care: protocol of the Quality indicators for Palliative Care (Q-PAC) study. BMC: Palliative Care. 2013;12(1):6.

39. Liu HC. A theoretical framework for holistic hospital management in the Japanese healthcare context. Health Policy. 2013;113:160-9.

40. Ludt S, Urban E, Eckardt J, Wache S, Broge B, Kaufmann-Kolle P, et al. Evaluating the quality of colorectal cancer care across the interface of healthcare sectors. PLoS One. 2013;8(5):e60947.

41. Mason C, Weber J, Atasoy S, Sabariego C, Cieza A. Development of indicators for monitoring Community-Based Rehabilitation. PLoS One. 2017;12(6):e0178418.

42. McCarthy M, Gonzalez-Izquierdo A, Sherlaw-Johnson C, Khachatryan A, Coleman MP, Rachet B. Comparative indicators for cancer network management in England: Availability, characteristics and presentation. BMC Health Services Research. 2008;8:45.

43. McEwan KL, Goldner EM. Keeping Mental Health Reform on Course: Selecting Indicators of Mental Health System Performance. Canadian Journal of Community Mental Health. 2002;21:5-16.

44. Meyer GS, Nelson EC, Pryor DB, James B, Swensen SJ, Kaplan GS, et al. More quality measures versus measuring what matters: a call for balance and parsimony. BMJ Qual Saf. 2012;21(11):964-8.

45. Murray SF, Buller AM, Bewley S, Sandall J. Metrics for monitoring local inequalities in access to maternity care: developing a basket of markers from routinely available data. BMJ: Quality & Safety. 2010;19(5):e39.

46. NHS [Institute for Innovation and Improvement], APHO [Association of Public Health Observatories]. The Good Indicators Guide: Understanding how to use and choose indicators. Guidance. Coventry, GB: NHS; [2007] First published: June 2007. Page updated: November 2017.

47. Parkinson J. Establishing national mental health and well‐being indicators for Scotland. Journal of Public Mental Health. 2006;5(1):42-8.

48. ni Riain A, Vahey C, Kennedy C, Campbell S, Collins C. Roadmap for developing a national quality indicator set for general practice. International Journal of Health Care Quality Assurance. 2015;28(4):382-93.

49. Schmitt J, Petzold T, Eberlein-Gonska M, Neugebauer EAM. Anforderungsprofil an Qualitätsindikatoren. Relevanz aktueller Entwicklungen der Outcomes Forschung für das Qualitätsmanagement. Z Evid Fortbild Qual Gesundhwes. 2013;107(8):516-22.

50. Smith KL, Soriano TA, Boal J. Brief Communication: National Quality-of-Care Standards in Home-Based Primary Care. Ann Intern Med. 2007;146(3):188-92.

51. Spaeth-Rublee B, Pincus HA, Huynh PT. Measuring Quality of Mental Health Care: A Review of Initiatives and Programs in Selected Countries. Canadian Journal of Psychiatry, The. 2010;55(9):539-48.

52. Stelfox HT, Straus SE. Measuring quality of care: considering measurement frameworks and needs assessment to guide quality indicator development. Journal of Clinical Epidemiology. 2013;66(12):1320-7.

53. Strömbeck B, Petersson IF, Vliet Vlieland TP. Health care quality indicators on the management of rheumatoid arthritis and osteoarthritis: a literature review. Rheumatology. 2013;52(2):382-90.

54. Van Damme N, Buijck B, Van Hecke A, Verhaeghe S, Goossens E, Beeckman D. Development of a quality of meals and meal service set of indicators for residential facilities for elderly. Journal of Nutrition, Health & Aging, The. 2016;20(5):471-7.

55. van den Driessen Mareeuw FA, Hollegien MI, Coppus AMW, Delnoij DMJ, de Vries E. In search of quality indicators for Down syndrome healthcare: a scoping review. BMC Health Services Research. 2017;17:284.

56. van Hulst LTC, Fransen J, den Broeder AA, Grol R, van Riel PLCM, Hulscher MEJL. Development of quality indicators for monitoring of the disease course in rheumatoid arthritis. Annals of the Rheumatic Diseases. 2009;68(12):1805-10.

57. van Lent WAM, de Beer RD, van Harten WH. International benchmarking of specialty hospitals. A series of case studies on comprehensive cancer centres. BMC: Health Services Research. 2010;10(1):253.

58. Veillard J, Champagne F, Klazinga N, Kazandjian V, Arah OA, Guisset AL. A performance assessment framework for hospitals: the WHO regional office for Europe PATH project. International Journal for Quality in Health Care. 2005;17(6):487-96.

59. Voerman GE, Calsbeek H, Maassen ITHM, Wiegers TA, Braspenning J. A systematic approach towards the development of a set of quality indicators for public reporting in community-based maternity care. Midwifery. 2013;29(4):316-24.

60. Wierenga PC, Klopotowska JE, Smorenburg SM, van Kan HJ, Bijleveld YA, Dijkgraaf MG, et al. Quality Indicators for In-Hospital Pharmaceutical Care of Dutch Elderly Patients. Drugs & Aging. 2011;28(4):295-304.

61. Wiles LK, Hooper TD, Hibbert PD, Molloy C, White L, Jaffe A, et al. Clinical indicators for common paediatric conditions: Processes, provenance and products of the *CareTrack Kids* study. PLoS One. 2019;14(1):e0209637.

62. Wollersheim H, Hermens R, Hulscher M, Braspenning J, Ouwens M, Schouten J, et al. Clinical indicators: development and applications. The Netherlands Journal of Medicine. 2007;65(1):15-22.
